# Food Innovation Adoption and Organic Food Consumerism—A Cross National Study between Malaysia and Hungary

**DOI:** 10.3390/foods10020363

**Published:** 2021-02-07

**Authors:** Robert Jeyakumar Nathan, Soekmawati, Vijay Victor, József Popp, Mária Fekete-Farkas, Judit Oláh

**Affiliations:** 1 Faculty of Business, Multimedia University, Jalan Ayer Keroh Lama, Melaka 75450, Malaysia; robert.jeyakumar@mmu.edu.my (R.J.N.); soekma_wt@yahoo.com (S.); 2 Department of Economics, CHRIST (Deemed to be University), Hosur Road, Bengaluru 560029, Karnataka, India; vijay.victor@christuniversity.in; 3 Faculty of Economics and Social Sciences, Szent István University, 2100 Gödöllő, Hungary; popp.jozsef@szie.hu; 4 TRADE Research Entity, North-West University, Vanderbijlpark 1900, South Africa; olah.judit@econ.unideb.hu; 5 Institute of Applied Informatics and Logistics, Faculty of Economics and Business, University of Debrecen, 4032 Debrecen, Hungary

**Keywords:** organic foods consumerism, food innovation adoption, food security, circular economy, health consciousness, environmental concern

## Abstract

In order to meet the rising global demand for food and to ensure food security in line with the United Nation’s Sustainable Development Goal 2, technological advances have been introduced in the food production industry. The organic food industry has benefitted from advances in food technology and innovation. However, there remains skepticism regarding organic foods on the part of consumers, specifically on consumers’ acceptance of food innovation technologies used in the production of organic foods. This study measured factors that influence consumers’ food innovation adoption and subsequently their intention to purchase organic foods. We compared the organic foods purchase behavior of Malaysian and Hungarian consumers to examine differences between Asian and European consumers. The findings show food innovation adoption as the most crucial predictor for the intention to purchase organic foods in Hungary, while social lifestyle factor was the most influential in Malaysia. Other factors such as environmental concerns and health consciousness were also examined in relation to food innovation adoption and organic food consumerism. This paper discusses differences between European and Asian organic foods consumers and provides recommendations for stakeholders.

## 1. Introduction

The human population is still growing fast. Today, the global population is around 7.8 billion. This number is expected to increase by 10% (8.5 billion) by 2030, 26% (9.7 billion) by 2050, and 42% (10.9 billion) by 2100, according to the U.N. Department of Economic and Social Affairs [1]. The growing population increases the demand for food, sometimes leading to the irresponsible use of natural resources which are becoming scarce [2]. This rising demand exerts pressure on the environment, resulting in massive deforestation and the deterioration in biocapacity and marine ecosystems [3]. Due to the emergence of biological hazards that affect quality of life, health concerns are more prevalent among consumers now than ever before. Meeting food supply challenges and feeding the growing global population with good quality food, has emerged as the new global food security agenda.

The increasing demand for organic food may reflect consumers’ concerns regarding the devastating effects of conventional agriculture on people’s health and the environment [2]. Rimal et al. [4] and Saba and Messina [5] found that consumers purchased organic food as they perceived that the risk of pesticide contamination is relatively low in organic food and the growing of organic food is perceived as harmless for the environment. Growing buyers’ interest in quality food, wellbeing, better health, and sustainable living make organic food a viable choice [6]. Organic food that is regulated is produced with less negative impact on nature as compared to the conventional food production process. The demand for organic food initiates the establishment of various organic farming techniques around the world, utilizing a minimum of synthetic inputs or none at all [7].

Besides health reasons, moral thought and responsibility towards the environment motivate some consumers to purchase organic food [8,9]. Hence, organic food has gained popularity and is seen as a way of life for some consumers [10]. Although organic food is positioned as a better food choice for health and the environment, the issue of its relatively higher cost is a hindrance to purchase for some. Surveys conducted in the United Kingdom, Japan, India, and Indonesia in 2015 revealed that consumers were willing to pay up to 30% more for fruits and vegetables as an act of social responsibility [11]. However, Timmins [12] noted that the advantages related to organic food were not sufficient for some purchasers to make the final decision to purchase organic food. Besides pricing concerns, the technology of producing organic food also draws consumer skepticism [13]. 

Overall, the demand for organic food globally is shaped by a number of economic, sociological, and psychological factors, which can vary from country to country and from type of commodity to food group [14]. Cross-national studies could aid in a better understanding global consumers’ similarities and differences and pave the way forward towards a more sustainable food and future for all. A recent cross-national study by Boobalan et al. [15] compared Indian and American organic food consumerism and found key differences between consumers in these two large countries regarding the psychological benefits they acquire when purchasing organic food. Against this backdrop, this study aims to investigate the factors contributing towards the intention of consumers from Asia (Malaysia) and Europe (Hungary) to purchase organic food, taking into consideration the role of the food innovation adoption behavior of consumers.

To this end, this paper is presented in sections, as follows. This introduction addresses the aim and focus of this research. Section 2 presents the literature review and subsequently the conceptual framework based on the critical secondary research review. This is followed by the description of the research methodology in Section 3. The research findings and discussion are highlighted in Section 4. Section 5 demonstrates the cross-national comparison of the findings between consumers in Malaysia and Hungary. Section 6 presents the conclusion of the study and Section 7 the limitations of the research and suggestions for future study. 

## 2. Literature Review and Theoretical Framework

According to the Oxford Dictionary, ‘organic’ simply means something that is derived from living matter. In the food and agricultural industry, the word ‘organic’ is a labeling term that is given by the regulators indicating the approval of methods for the production, handling, and processing of organic foods sold. Organic food cultivation integrates cultural, biological and mechanical practices that lead to resource conservation and recycling of resources which promote ecological balance and biodiversity conservation [16].

In Malaysia, organic certification is regulated under the Malaysian Quality Standard 1529:2015 which ensures that the practice of organic farming is based on the four principles of Health, Ecology, Fairness and Care. The Malaysian organic standard emphasizes the health of soils, plants, animals, and humans, and the well-being of the ecological system, the environment, as well as balance and fairness to the ecological system [17]. In Hungary, the procedures of organic products’ certification, production, labeling and marketing are governed by the [18]. The EU Regulation 2018/848 (Article 1) describes organic production as *“an overall system of farm management and food production that combines best environmental and climate action practices, a high level of biodiversity, the preservation of natural resources and the application of high animal welfare standards and high production standards in line with the demand of a growing number of consumers for products produced using natural substances and processes. Organic production thus plays a dual societal role, where, on the one hand, it provides for a specific market responding to consumer demand for organic products and, on the other hand, it delivers publicly available goods that contribute to the protection of the environment and animal welfare, as well as to rural development”.*

‘Organic labelled’ foods are produced without the use of pesticides and artificial nitrogen composts, antibiotics, synthetic hormones, genetic engineering, or other detrimental practices prohibited in the regulation [19]. The entire organic food value chain is regulated to ensure that it is environmentally safe and free from irradiation, industrial solvents and synthetic food additives [20]. Based upon the stringent regulatory framework for producing organic food, the ‘organic’ label thus gives assurance to buyers that it the food is produced without harming the environment and without chemical residues in food. It serves as an assurance that the food is free from toxic and harmful substances. 

To obtain an organic certification, farmers need to ensure that their fields are processed naturally, and free from prohibited materials for at least three years [19], as healthy soil has a profound impact on the quality of crops. Organic farmers are also expected to use ethical practices in farming such as hand weeding, mulching, intercropping, using mechanical control against pests, spread yields, crop revolution, and thick planting, instead of using conventional pesticides, herbicides, and engineered nitrogen manures, in order to enhance soil health [21]. 

There is an increased interest in the study of organic food production as it is also linked to food security and the sustainable supply of food to promote the circular economy. Previous studies have shown that consumers were motivated to purchase organic food due to health and environmental concerns [20,22,23]. Studies also found that consumers’ health consciousness predicted their consumption of organic food [24,25,26]. Subjective norms including the influence of family and friends, compounded with lifestyle trends, also show a significant influence on the intention to purchase organic food [27,28].

In this cross-national study, five determinants of organic food consumerism were measured to assess their impacts on consumers’ purchase intention towards organic food. These factors were found to have common interest in research into organic food consumerism for both European and Asian consumers in recent literatures. The first four factors are health consciousness, environmental concern, perceived quality of organic food, and social lifestyle factors. The fifth factor that this study introduces to the literature is the impact of consumers’ adoption of food innovation technologies on their organic food purchase intention. Food innovation adoption is introduced as both an independent variable and a mediating variable in this study in order to examine its wider role in organic food consumerism. 

### 2.1. Organic Food Consumption Trends in Hungary and Malaysia

Despite the excellent agricultural conditions in Hungary and Malaysia, the proportion of land used for organic production is relatively low compared to conventional farming (4.0% of total agricultural land in Hungary according to the Central Statistical Office [29] and 0.1% in Malaysia according to Willer et al. [30]). Consumer spending on organic food is still lower than conventional food products and it is believed that by increasing the demand for organic food, better food sustainability can be achieved via a transformation of the food value chain [30]. 

Within the Asia-Pacific region, people consume organic produce because of its health benefits and its advanced biological farming techniques. Demand for, and the consumption of, organic foods and beverages in the Asia-Pacific region are predicted to grow from 2020 to 2025 [31]. In the Asia-Pacific region, Malaysia is one the countries offering great opportunities for organic food to flourish. Recently, Malaysian shoppers have become more cognizant of well-being, and hence have increased their consumption of organic substitutes for conventional food. Nevertheless, the supply of organic produce in Malaysia is unable to meet the local market demand, causing a nationwide shortage of organic food. Malaysia still vigorously imports organic food from Europe and North America [32]. In Europe, Christos and Athanasios [33] predicted a lack of supply of organic food, not a lack of demand for it. 

The United States recorded the largest sales of organic food (43%) in 2017, followed by the European Union member states (38%) [34]. Among Central and Eastern European countries, Hungary ranked as the third largest market volume of organic foods in 2010 [35]. Hungary is among the largest exporters of organic food in Central Europe. In 2018, there were 3929 producers who cultivated a total of 209,382 hectares of organic-farming land in Hungary [36]. 

As regards the domestic consumption of organic food, studies found that European consumers were driven by health consciousness, environmental concern, quality of life, and technological development [24,37]. Although environmental and health consequences can influence organic consumerism, the affordability of organic food was significant in determining consumers’ food choices, particularly those in Italy and Hungary [25]. In Hungary, innovation in the food industry has been evaluated favorably by consumers [38]. This implies the significance of technological innovation in the food industry in satisfying consumers’ needs [38]. Interestingly, food-technology was found to be related primarily to environmental concern among Hungarian consumers [39]. Similarly, in Malaysia, the creation of organic food has turned into an inventive methodology for the food sector to meet the rising consumer demands for healthier food choices. 

### 2.2. Consumers’ Purchase Intention towards Organic Food

Consumers’ purchase intention is explained simply as the possibility that a consumer will acquire a product [22]. This variable is used in social science and business literature to indicate the actual consumption behavior of consumers towards a product or service [40]. It represents the likelihood that a purchase would take place as a result of “the interaction between customer needs, attitude and perception towards the product” [41]. Purchase intention acts as a measure of consumers’ attentiveness in acquiring a product and the possibility of actually purchasing it [42]. According to Park and Kim [43] and Shin [44], purchase intention can be treated as a predictor of the actual purchasing decision due to its inclination to approximate to the actual conduct of a consumer. Although having an intention to purchase would more likely lead to actual purchase, it cannot be assumed that all predictors used would lead to actual purchasing action. Behavioral intention is formed based on an individual’s motivation to perform that behavior, taking into account alternative options and his or her currently active goals [45]. With the limitation of observing the actual purchase behavior of consumers, purchase intention is used in this study to measure the potential of consumers’ purchases.

Gifford and Bernard [46] employed a two-limit Tobit model and found that purchase intention towards organic foods among consumers may be influenced by the perceived benefits of organic agricultural methods, and the perceived risk of purchasing food grown using conventional procedures. In addition, Verhoef [47] posits that consumers are not only motivated by their rational economic motives, but also by emotional motives when purchasing organic food. The study found that consumers were willing to pay premium prices for organic food due to emotional motives, such as fear, guilt and empathy towards the environment.

Based on the relevant previous works, this study identified five variables to form an organic consumerism framework to compare Malaysian and Hungarian consumers as regards their organic food purchase behavior. They comprise food innovation adoption, health consciousness, environmental concern, perceived quality, and social lifestyle.

### 2.3. Health Consciousness

Health consciousness means that an individual’s orientation toward his or her efforts to prevent illness and improve overall well-being [48]. Iversen and Kraft [49] defined health consciousness as “a tendency to focus attention on one’s health” (p.603). An individual’s level of health could be assessed through how one searches for health information and incorporates it into daily life. Homer and Kahle [50] posit that there is a relationship between consumers’ intrinsic motivation, such as self-fulfillment, and a sense of accomplishment in purchasing nutritional food.

Health-conscious consumers are cognizant of their wellness and this health concern drives them to continuously improve their health and quality of life. To measure health consciousness, Ellison et al. [51] used behaviors such as food consumption, exercise, and substance use as indicators. Since the concept of health consciousness is linked more to personal attributes, measuring one’s health consciousness on a psychological basis would better predict diverse health behaviors and result in greater construct validity.

Health consciousness has been relevant in predicting purchase intention and behavior regarding organic food production since buyers are aware that their food intakes impacts on their health. Previous research done by Shaharudin et al. [52] identified that consumers’ attention to their health was a primary motive for the purchase of organic food. From another study, 87% of consumers believed that organic food was a healthier choice as compared to conventional food [10]. Similarly, Michaelidou and Hassan [53] highlighted health consciousness as the most important motive in explaining consumers’ attitudes and behavior towards organic foods. 

Shaharudin et al. [52] found the most popular motive to purchase organic food was consumers’ perception of organic food as a healthier option for them. They also identified that consumers’ interest in health was their primary motive to purchase organic food. Although the inherent evidence of the health benefits of consuming organic food have not been validated by Meemken and Qaim [2], a positive relationship between consumers’ health consciousness and their purchase intention has been frequently identified in previous studies. Thus, this study hypothesized that health consciousness would positively influence consumers’ intention to purchase organic food (Hypothesis 1 (H1)).

### 2.4. Environmental Concern

Consumers who are environmentally conscious prefer to use certain products because they believe they can reduce ecological impacts [54]. Similarly, consumers of sustainable wines were willing to change their consumption behavior to minimize the negative impact on the environment [55]. This type of consumer, also referred to as green consumers, often determine their purchase behavior for the benefit of the environment. The more consumers are concerned about the environment, the more positive are their attitudes toward organic food [56].

Seventy-five percent of respondents in the study by Petrescu and Petrescu-Mag [8] believed that organic food contributes to environmental protection. Congruently Basha et al. [57], found consumers’ attitude towards purchasing organic food was strongly influenced by their concern for the environment. Sogari et al. [55] investigated consumers’ environmental concerns and their intention to purchase sustainable wines and found it was important for consumers to believe that sustainable wines truly benefitted the environment in order to form a positive attitude towards purchasing sustainably.

In this study, a positive impact of consumers’ environmental concern on their intention to purchase organic food is presented for testing in Hypothesis 2 (H2).

### 2.5. Perceived Quality

Perceived quality has gained popularity in marketing studies as a predictor of purchase intention and consumers’ satisfaction. It is considered a crucial key for business sustainability, especially in competitive markets [58]. Perceived quality is defined as the personal judgment of the quality and benefit of a product or service that consumers establish in their minds [26]. The value of a product, also known as product utility, is often evaluated based on its ability to meet consumers’ needs, resulting ultimately in their satisfaction. Consequently, the higher the value a product has is in consumers’ minds, the higher the price they are willing to pay for it. 

Consumers who purchase organic food often appear to be particularly concerned about the quality of the foods they consume. Half of the consumers who participated in a survey conducted by Timmins [12] agreed that organic food had better quality and taste. However, the major barrier to organic consumption was still the higher price. Some consumers perceived that the benefits of organic foods were not sufficient to justify its higher price [12]. Although value-for-money is found to be important for some consumers, one previous study finds this does not translate into anti-organic attitudes [12]. The affordability of organic foods played a major role in influencing consumers’ food selection, particularly those in Hungary [25]. 

The locality of the organic food supply could potentially off-set the high price concern linked to organic foods. Consumers believe that locally produced greens produce a smaller carbon-footprint and are thus more environmental friendly and sustainable [59,60]. Timmins [12] found that 60% of his respondents were interested in locally sourced crops. Although affordability could influence consumers’ food selection, the perceived quality of organic foods was found to be significant in predicting consumers’ purchase intentions. This study predicts a positive relationship between perceived quality and consumers’ intention to purchase organic foods (Hypothesis 3 (H3)).

### 2.6. Social Lifestyle

Studies in psychosocial theories and health behaviors explore how cognitive and social factors affect human health and disease [61]. Social and lifestyle factors relate to how peers and the people who surround a person affect his or her decision making. Additionally, messages through the media as well as reference groups and celebrities can also influence an individual’s decision making [45]. Previous studies have shown the strong impacts of social factors on an individual’s decision making in a wide variety of situations including business, social and health decisions [62,63,64].

Petrescu and Petrescu-Mag [10] explain the positioning of foods as fashionable items and their consumption as a social phenomenon that can generate consumers’ interest and in turn become a part of their lifestyle. The trend and image factors may also influence consumers’ decision to purchase organic foods despite the higher price. For instance, trendsetters in Vietnam who enjoy cooking pay greater attention to healthy food and prefer organic foods [27]. Specifically, a study involving youngsters by Vermeir and Verbeke [28] found a strong impact of social influence on sustainable food consumption behavior among young adults in Belgium. 

The media often broadcasts programs showing the enjoyment of food and cooking in such a way that boosts the importance of food in representing power, pleasure, cleverness, and beauty. Often, people strongly believe that “who you are” to some extent is reflected in “what you buy”. Social status was often found to be a determinant influencing people’s decisions to consume green products rather than their more luxurious, non-green counterparts [65]. Similarly, Sahelices-Pinto et al. [66] showed that the consumption of organic foods was influenced by both social factors and self-esteem, revealing the impact of organic consumption on boosting one’s social identity. Thus, hypothetically, a positive relationship may be established between social lifestyle and consumers’ intentions to purchase organic foods (Hypothesis 4 (H4)).

### 2.7. Food Innovation Adoption

Food security has become a vital point of focus globally [67,68]. It is included as being of paramount importance in the United Nation’s Sustainable Development Goals, as Goal 2 [69]. Goal 2 calls on all the nations of the world to work together to end hunger, achieve food security and improve nutrition, and promote sustainable agriculture. Altogether, the SDG Goal 2 proposes 8 targets to be achieved globally by the year 2030. The third target in the goal (Target 2.3) aims to double agricultural productivity, while the sixth target (Target 2.a) specifically mentions increased investments in technology development. In order to meet these two targets, the pivotal role of technology and innovation in food production is highlighted. 

Fortunately, innovations in digital technologies such as advanced data analytics, predictive modeling, robotics, and the Internet of Things (IoT) have increased the efficiency of modern farming. Biotechnology advances in food technology also assist in increasing the food supply. By utilizing food innovation technologies that provide timely and accurate data, farmers can significantly improve their farming processes and eventually improve productivity. The new application of digitization and IoT in farming makes it possible to assess the soil moisture level, temperature, and many more agricultural matrices in real time to facilitate farmers’ timely and accurate interventions. 

The EU Regulation 2018/848 (Article 24) reads: “In order to support and facilitate compliance with this Regulation, operators should take preventive measures at every stage of production, preparation and distribution, where appropriate, to ensure the preservation of biodiversity and soil quality, to prevent and control pests and diseases and to avoid negative effects on the environment, animal health and plant health. They should also take, where appropriate, proportionate precautionary measures which are under their control to avoid contamination with products or substances that are not authorized for use in organic production in accordance with this Regulation and to avoid commingling organic, in-conversion and non-organic products”. Based on this article, organic farmers can still use preventive measures to ensure their crops are safe from pests and diseases. However, if unauthorized substances are used in any of these activities, the products can no longer be considered organic. Hence, technology-based preventive measures would be ideal in order not to contravene this article and lose the organic product label. 

As consumers’ food preferences move towards fresh and whole foods, food-processing technology is also forced to meet the highest environmental standards with minimal alteration in the qualities and original flavors of the foods. As a result, organic farmers and their distributors spearhead the trend towards sustainable food production and a more transparent value chain [70]. This move towards a sustainable cycle of production is also referred to as the circular economy where the main goal is to reduce waste in the food production lifecycle [71]. 

The adoption of food innovation technology may influence consumers’ purchase of organic foods, as food innovation technologies are rapidly being introduced into organic farming. Accordingly, this study proposes the fifth hypothesis to measure the impact of food innovation adoption of consumers on their intention to purchase organic foods (Hypothesis 5 (H5)).

### 2.8. Food Innovation Adoption Behaviour as Mediator in Organic Foods Purchase Intention

It is crucial for the food sector to identify the important drivers of consumers’ preferences for foods in these modern times [72]. Consumers have become increasingly conscious of what they eat for various reasons, including skepticism as to whether food technology really produces better quality foods that warrant the higher price. As the biggest stakeholder in the food supply chain, consumers’ preferences and decision making in foods purchase make them a formidable force for the food industry to reckon with. Mindful consumers are looking for the move towards sustainable food production. Health consciousness, environmental concern, perceived quality of organic foods, and social lifestyle would hypothetically impact their food innovation adoption behavior. 

This study postulates that food innovation adoption would have both a direct impact on the intention to purchase organic foods (Hypothesis 6, 7, 8, and 9), and mediate the impact of health consciousness, environmental concern, perceived quality and social lifestyle on consumers’ intention to purchase organic foods (Hypothesis 10, 11, 12, and 13). 

The following list presents Hypotheses 6 to 13 which are put forward for testing in this study:

**Hypothesis 6** **(H6).** *There is a positive impact of consumer health consciousness on food innovation adoption.*

**Hypothesis 7** **(H7).** *There is a positive impact of consumer environmental concern on food innovation adoption.*

**Hypothesis 8** **(H8).** *There is a positive impact of consumer perceived quality of organic food on food innovation adoption.*

**Hypothesis 9** **(H9).** *There is a positive impact of consumer social lifestyle on food innovation adoption.*

**Hypothesis 10** **(H10).** *The impact of health consciousness on consumers’ purchase intention is mediated by food innovation adoption.*

**Hypothesis 11** **(H11).** *The impact of environmental concern on consumers’ purchase intention is mediated by food innovation adoption.*

**Hypothesis 12** **(H12).** *The impact of perceived quality on consumers’ purchase intention is mediated by food innovation adoption.*

**Hypothesis 13** **(H13).** *The impact of social lifestyle on consumers’ purchase intention is mediated by food innovation adoption.*

**Figure 1:**
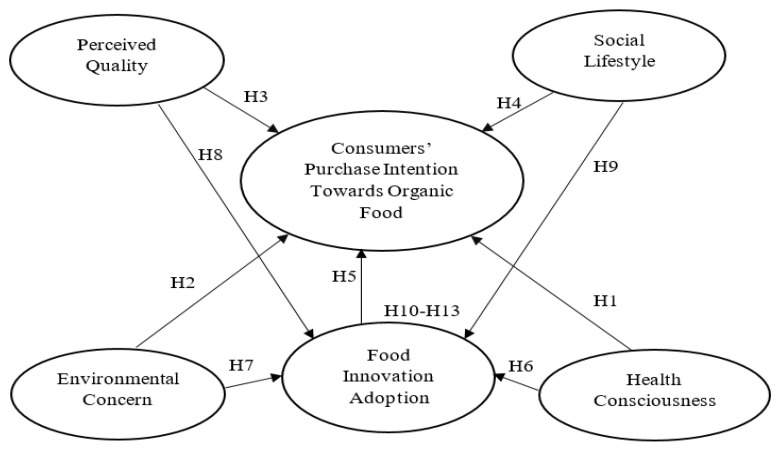
Research theoretical framework.

The research variables and corresponding hypothesis are shown in Figure 1.

## 3. Methodology

According to Roitner-Schobesberger et al. [73], there have been numerous debates on buyers’ views of organic foods in the United States and Europe; however, less has appeared in Asia despite the growing market for organic foods. For this reason, an analysis of organic foods consumerism in Malaysia—one of the leading contributors of agriculture in Asia—is selected for this study. Hungarian consumers in this study were chosen to represent organic consumerism in Europe. Although at this juncture, this comparison does not provide a holistic comparison between European and Asian consumers, in this pioneering cross-national study of organic foods consumerism, these two countries were chosen due to the proximity of the researchers to both countries to facilitate insightful data collection and to provide preliminary insights into this area of research. The findings of this research could warrant more comprehensive work in the future between Asia and Europe.

To conduct a cross-national comparison analysis of organic food consumerism in Malaysia and Hungary, this study utilized a research questionnaire as the data collection instrument for gathering primary research data. The participants in this study from both countries were approached randomly using the purposive sampling methodology and the classic mall-intercepted survey technique. The availability of organic food products in the areas where the respondents were approached was confirmed before administering the questionnaire to potential participants. Only participants who had prior experience of purchasing organic foods were selected as respondents. The survey was administered face to face among respondents in Malaysia and Hungary. Hardcopy questionnaire forms were used for data collection, which was carried out between June 2019 and March 2020 in both countries. 

In total, 300 usable responses were obtained in Malaysia and 372 in Hungary. The filled questionnaires were carefully screened for missing data and mistakes in responses such as multiple responses for single response questions. Verified questionnaires were coded in the statistical software IBM Statistical Package for Social Sciences (SPSS), version 27, for descriptive analysis. Hypothesis testing and path modelling was done using Partial Least Square Structured Equation Modelling (PLS-SEM) using ADANCO PLS Software, Version 2.0. PLS-SEM was selected as the data analysis technique as the research model of this study is geared towards predictive modelling and testing the relationship between new constructs. Kline [23] pg. 286 recommends PLS-SEM as “well suited for where: (1) prediction is emphasized over theory testing and (2) it is difficult to meet the requirements for large samples or identification in SEM.” Based on these criteria, the PLS-SEM technique was selected as the appropriate technique for hypotheses testing and path modelling for this study.

All measurement items of the research variables were measured using a five-point Likert scale based on the extent to which respondents agree or disagree with the particular indicator (item) statement in the questionnaire on a scale of 1 to 5; where (1) is Strongly Disagree, (2) is Disagree, (3) is Neutral, (4) is Agree, and (5) is Strongly Agree. This scale design is commonly used as measurement for social science studies. Churchill and Iacobucci [20] noted questionnaires using the Likert scale could provide appropriate measurements that would ease the process of tabulation and statistical analysis.

The indicators for Food Innovation Adoption (FIA), were self-developed for this study. These indicators were expert reviewed by two professors at the Multimedia University, Malacca Campus, Malaysia, who are specialists in technology adoption studies. Furthermore, the indicator statements were validated through a pilot study with data collected at the Multimedia University Malacca Campus among undergraduate students. The data from 200 samples showed the high reliability and internal consistency of the self-developed indicators, hence the indicator statements were incorporated in the final questionnaire for the productive phase of data collection in Malaysia and Hungary.

## 4. Results

Table 1 shows the respondents’ demographic details.

Of the 300 respondents in Malaysia, 59% were females, and 71.3% were between 21 and 40 years old. Most of the respondents (83%) were single. In Hungary, 60.2% of the total respondents were males. As for ages, they had almost an equal number of respondents who were 21–40, 41–50 and 51–60 years old, with 36.8% between 21 and 40 years old. Lastly, more than half of the respondents in Hungary were married with children (53.2%), while in Malaysia this figure was about 15%.

Table 2 shows the research variables, indicator sources, aggregate means and standard deviations for both Hungarian and Malaysian data.

The measurement model is assessed via construct validity, convergent validity, and discriminant validity analyses. Before conducting hypotheses testing, it is essential to investigate the indicators’ factor loadings. According Hair et al. [77], indicators with loadings below 0.50 should be removed from the path model due to the low predictability of the relevant variable. Thus, HC5, EC4, PQ5, SL1, FIA1, FIA3, and CP5 were removed from both the Hungarian and Malaysian path models in order to make identical comparison of path modelling for both countries (refer to Table 3).

For factor loadings that were above 0.50 but below 0.70, their variable’s composite reliability (CR) and AVE are confirmed to exceed thresholds of 0.70 and 0.50 (Hair et al. [77] and Bagozzi and Yi [78]), assuring the path models’ Reliability and Convergent Validity. As for the Cronbach Alphas, all values are above 0.70, fulfilling the satisfactory values, except for SL 0.673 (Malaysia) and 0.671 (Hungary), which were slightly below the 0.7 threshold; however, their CR and AVE are above threshold levels, hence fit for path modelling [79]. The statistics of all constructs and indicators are presented in Table 3.

A high inter-relationship and multi-collinearity between variables can lead to misleading findings, magnified standard errors, or weaker power of regression coefficients. According to Henseler et al. [80], when all the values of the Heterotrait-Monotrait Ratio of Correlations (HTMT) are lower than 0.85, this implies that the variables are conceptually distinct from each other. HTMT 0.85 is used in this study as the conservative criterion to assess discriminant validity [80]. From Table 4, it is observed that all HTMT values among the variables in this study are lower than the thresholds of 0.85, indicating the models are free from multi-collinearity.

Additionally, to assess the goodness of fit of the research model, the Standardized Root Mean Square Residual (SRMR) was calculated. The results show SRMR values of 0.0670 for Malaysia and 0.0541 for Hungary. The SRMR values for the Malaysian and the Hungarian models are within the threshold level of 0.08 (Hu and Bentler [81]), assuring the goodness of fit of the research models for both countries. The R square values for Malaysia are CP = 0.408 and FIA = 0.458; while R square values for Hungary are CP = 0.725 and FIA = 0.493. The research variables show a high variance explained in both models; especially with the Hungarian consumer purchase intention of organic foods, the model shows that the research variables account for approximately 73% of the variance. 

### Hypotheses Testing

For testing the hypotheses, bootstrapping with 5000 iterations was applied. The significance of the path coefficient is assessed to validate each hypothesis. The structural model for Hungary and Malaysia with the R square values, path coefficients, and factor loadings are presented in Table 5.

Based on the results of the Malaysian and Hungarian path analysis, it was found that the relationships between Health Consciousness (HC) and Environmental Concern (EC) regarding Consumer’s Purchase Intention Towards Organic Foods (CP) are insignificant for Malaysia; however, these paths are significant for Hungary (Hypotheses 1 and 2 are partially supported—true only for Hungary). Perceived Quality (PQ), Social Lifestyle (SL) and Food Innovation Adoption (FIA) each show a significant impact on CP in both countries (Hypotheses 3, 4 and 5 are supported). 

Among these five independent variables (H1 to H5), SL has the highest impact on CP (0.306) for Malaysia. However, for Hungary, FIA shows the highest impact on CP (0.414). It is interesting to note that the same factor (FIA), though significant, shows the lowest impact on CP in the Malaysian context (0.109). This indicates the difference in perception and role that FIA plays in these two countries. 

On the other hand, PQ is found to be the second most important factor leading to CP in Malaysia and in Hungary. This finding highlights the consistent perception of users in both countries who tend to relate the perceived quality of organic foods with their purchasing intention. 

As for the impact of the research variables on FIA as mediating variables, HC, PQ, and SL were found to be significant predictors of FIA in Malaysia and Hungary (Hypotheses 6, 8 and 9 are supported). Testing the impact of EC on FIA shows differing results for the two countries, where EC on FIA is significant in Malaysia but not in Hungary (H7 is partially supported). This shows that although EC leads to CP in Hungary, it does not significantly predict Hungarians‘ FIA behavior.

To further investigate the mediating effect of FIA in the relationships between each of the predictors HC, EC, PQ, and SL to CP, the significance of these indirect paths was tested. The results of the indirect effects are presented in Table 6.

The result of the indirect effects analysis reveals several significant paths. According to Hair et al. [77], it is necessary to evaluate indirect effects in order to determine whether a mediating effect is present. When both direct and indirect effects are significant, a partial mediation is observed; if the indirect effect is significant but the direct effect is insignificant, a full or indirect-only mediation is identified. However, when the indirect effect is insignificant, but the direct effect is significant, it indicates that a direct-only effect or no mediation effect is present [77].

From the mediation results above, it is observed that FIA is a significant mediator for EC and SL impacts on CP for Malaysia. However, it is not a significant mediator for PQ and HC. Reading this finding together with the earlier finding of the direct effect of HC on CP, it was also found not to be significant for Malaysia, while the direct effect of FIA on CP was significant. From these three findings, it can be deduced that for Malaysian consumers, health consciousness is an important reason that makes them consider accepting innovation in food production; however, health consciousness in itself is not the reason for purchasing organic foods. 

As for the Hungarian data, the finding shows FIA as a significant mediator for HC, PQ and SL on CP. However, FIA is found not to be a mediator for EC. Compounding this finding with the direct impacts of EC on CP (significant) and FIA (not significant), it can be deduced for Hungarian consumers that environmental concern is an important factor of consideration for them when purchasing organic foods; however environmental concern in itself is not a reason for adopting innovation in food production. Based on this finding, Hypotheses 10, 11 and 12 are partially supported, while Hypothesis 13 is fully supported.

## 5. Discussion

The data obtained in both countries revealed that consumers in both countries have some commonalities and some key differences in their adoption of food innovation, as well as in the purchase of organic foods. This section presents a critical discussion of these findings for Malaysia and Hungary. 

When it comes to the purchase of organic foods, both countries show different crucial determining factors that affect their decision making. To assist with visualizing the findings, Table 7 is based on the statistical results of path modelling coefficients in Table 5.

For Malaysian consumers, SL is the most crucial factor, followed by PQ and FIA, in determining their organic foods purchase intention. Comparing this with Hungarian consumers, the result shows that FIA is the most crucial determinant for Hungarians, followed by PQ, HC, EC, and finally SL.

The social lifestyle factor is found to be the most important factor that contributes to the intention to purchase organic foods in Malaysia. The social lifestyle variable measures buyers’ concerns regarding status and peer influences. Malaysian consumers demonstrate a greater tendency that social lifestyle will be a reason to purchase organic foods, which are more expensive than conventional foods. Social influence was also found in previous studies to impact on consumers’ intention to purchase organic foods in Malaysia by Ayub [77,82] and in Pakistan [83]. In particular, peer pressure was found to be a significant determinant in persuading young Malaysian consumers to purchase green products in a previous study [84]. Malaysian consumers appeared to purchase organic products with the intention of fulfilling and expressing their social identity [85] which is found to be consistent with the findings of this study.

Other findings from the region, such as Nguyen et al. [86], reveal that organic foods label significantly contributed to buyers’ favorable attitude to buying organic foods among urban Vietnamese consumers, while Fogarassy et al. [71] found that highly educated young people who are very conscious and live on good incomes may be the target group for circular innovation in Hungary. The study found that young consumers, the internet savvy, and software users living in cities buy organic foods and follow healthy lifestyle trends. Hence, having access to a more expensive food selection may be seen as a social symbol and a differentiator from the masses, as well as a sustainable lifestyle trend. 

The perceived quality of organic foods is found to be the second most important factor that drives the intention to purchase organic foods in Malaysia. Malaysian consumers seem to compare conventional foods with organic foods based on this perceived main difference—its quality. In previous studies by Lee and Yun [87] and Lockie et al. [88], consumers were found to be committed to foods they perceived to be natural, nutritional and free of unnecessary processing as well as artificial additives. Organic plants contain lower levels of pesticide residues and minimum concentrations of nitrate and cadmium. Besides, organic animal products were also found to contain higher levels of omega-3 fatty acids. Overall, organic foods were associated less with allergies, eczema, and obesity. Although there was insufficient evidence to draw conclusions on the positive health outcomes of consuming organic foods in the study by Meemken and Qaim [2], the study found that consumers from Malaysia and Hungary do associate organic foods with higher quality. 

Although health consciousness was expected to be an important reason for purchasing organic foods, this finding is contrary to the conventional wisdom. According to [84,89], health concerns were found to be more important than environmental issues for Indian consumers while they make purchasing decisions for organic foods. However, this study finds Malaysian consumers do not significantly associate health consciousness with their intention to purchase organic foods, but they do associate health consciousness with food innovation adoption, which is an important finding. FIA seems to fit the missing piece of the puzzle, in that it explains the inter-relationship between the health consciousness of consumers and their intention to purchase organic foods, as a mediator. 

Food innovation adoption is the most crucial reason for the intention to purchase organic foods in Hungary. Hungarian consumers seem to show greater awareness of food innovations compared to Malaysian consumers. This is perhaps due to the greater usage of technology in the agricultural sector in Hungary and Europe in general, as compared to Asia where most countries still rely on human labor for agricultural output [90,91,92]. The labor intensity in Asian agricultural production could also be related to the type of crops they harvest. Rice cultivation is purportedly more labor-intense as compared to wheat production, contributing to the greater demand for human labor in Asian agriculture (Vollrath [93]). 

A lesser emphasis on human labor in agriculture possibly allows European countries such as Hungary to focus more on food innovation technology. As a result, both capacity and performance in ecological innovations are found to be better in European countries, as compared to Asian countries [94]. The Hungarian data analysis shows the distinctly high impact of FIA (Beta Coefficient = 0.414) on consumers’ intention to purchase organic foods. While FIA is a third important factor for Malaysian consumers, this finding shows a significant difference between European (Hungarian) and Asian (Malaysian) consumers. Food innovation adoption is an important determinant of intention to purchase organic foods among buyers in Europe, but not a strong determinant in Asia. 

Although environmental concern was significant in determining Romanian consumers’ eating habits (Oroian et al. [95]), this study finds environmental concerns do not have a substantial effect on Malaysian consumers’ intention to purchase organic foods, and it is also the second least important factor that predicts the intention to purchase organic foods in Hungary. This inferior result of EC could be due to current consumers’ motives in consuming organic foods, which is not primarily driven by their intention to protect the ecological environment. Rather, their motives are based on social lifestyle factors and perceived quality (for Malaysia) and food innovation, perceived quality, and health concerns (for Hungary). This finding is consistent with recent research that found health factor and maintaining social status in society take priority in consumers’ minds over environmental safety [96].

The results for FIA reveal peculiar findings. EC is the most crucial determinant of FIA in Malaysia, while it is not significant in Hungary (but significant on CP in Hungary). Environmental concerns or ecological consciousness are seen as important determinants for FIA among Malaysians. There is a strong association between environmental protection and food innovation technology in Malaysian consumers’ minds. These two dimensions are seen as highly connected. Conversely, for Hungarians, EC is seen as a ‘distant factor’ that has no direct impact on their food innovation adoption behavior. EC, although significant for Hungarians in their organic food purchases, is not something they associate with FIA. Perhaps Hungarian food consumers do not look at innovation in food technology as something that is truly protective and conserving the environment. This suggests a possible skepticism towards food innovation technology and production, which are perhaps not viewed as environmentally friendly albeit perceived to be producing good quality foods [97,98]. It is worth noting this major difference between Asia (Malaysian) and Europe (Hungarian) where Asian food consumers in this context associate environmental concerns with FIA, while European consumers seem not to associate the two. 

Although Hungarian consumers do not associate EC with FIA, they strongly associate SL with FIA. Although social factors and status were not strong determinants of their intention to purchase organic foods, they are significantly more important in their FIA. This suggests Hungarian consumers consider social and lifestyle factors as trends that go together with innovation in the food sector. This possibly indicates that in their mind, innovation in food technology is just another social and lifestyle trend [99]. Social lifestyle trends are also found to be equally important elements in the Malaysian context which drive their adoption of food innovation. This could indicate a global trend of innovation in food technology being perceived by consumers as a social and lifestyle trend.

Hungarian consumers also show the high impact of PQ on CP, which indicates their high trust in food innovation technologies which are perceived to produce high quality foods, although they were skeptical about the environmental impact of FIA. 

It was considered meaningful to include food innovation adoption as an important construct in the modelling of this study and to provide an understanding of the wider ecosystem of organic food consumerism. The indirect effect results show that food innovation adoption seems to significantly mediate the relationships between the independent and dependent variables for both countries in most relationship paths. For Malaysian consumers FIA effectively mediates the impact of EC and SL on CP, while for Hungarian consumers FIA effectively mediates the impact of HC, PQ and SL on CP. For an elusive construct such as the FIA which had previously been less understood, the findings of this research show its pivotal role in understanding the ecosystem of organic food consumerism.

## 6. Conclusions

Consumer consciousness towards a more natural lifestyle and consumption behavior has led to various attempts to incorporate technology and trends in food production innovations. Various studies have discovered that buyers were increasingly troubled about the kind of foods they consume daily [100,101]. The rising interest in nutritious foods is reflected in consumers’ demand for organic food alternatives that promise better quality foods through the innovative use of technology and innovation in production. Food innovation serves the twin-role of providing high quality foods, as well as increasing the production of foods to meet rising global food demand.

Cross-national studies are gaining popularity as they are meaningful ways to provide new insights into consumer behavior by comparing consumer choices and actions in different cultures [87]. To add to this body of literature, this study measured important factors that influence the intention to purchase organic foods in Hungary and Malaysia, both countries that are strong in agricultural output in their regions. Additionally, this study identified food innovation adoption as an important variable to be included in the model as evidenced in recent food technology literatures, to better understand the organic food consumerism ecosystem impacted by food innovation technologies. We found food innovation adoption plays a critical role in explaining consumers’ organic foods purchasing behavior in Hungary and Malaysia.

The marketing of organic foods could emphasize the quality of organic foods as this is found to be the biggest driver of the intention to purchase organic foods in both countries. Social and lifestyle factors are highly significant in driving purchasing intentions. Consumers associate organic foods with trends in society and see it as a lifestyle choice. This could be a persuasive narrative for governments, policy makers, organic food producers, and retailers in improving engagement with consumers to promote sustainable consumption behavior. This could also lead to greater involvement of organic food buyers in the organic foods value chain, which is desirable for consumers [102]. Organic food growers and retailers may provide more information and transparency regarding their cultivation process, which is often invisible to final consumers. This lack of transparency may be leading to skepticism towards food innovations that are utilized in the production of organic foods. 

## 7. Limitations of the Study and Future Directions

Although the sample size obtained in this study was statistically significant, the demographics of the respondents from both countries were not similar. A more proportionate sampling of respondents based on national population statistics may provide more comparable data. Future studies could investigate demographic control variables as well as assess their moderating effects on food innovation adoption and the intention to purchase organic foods.

The purposive sampling methodology was used to select respondents in this study, due to the absence of a sampling frame. Future studies could collaborate with retailers to create a list of organic foods purchasers through customers’ purchase records to target actual customers who have purchased organic foods to be included for data collection.

This study is also limited in measuring the consumer purchase behavior related to organic foods. We used purchase intention as a measure to estimate actual behavior. Future studies can address this limitation by measuring the actual purchase of organic foods.

There seems to be a higher level of skepticism, especially in Europe, regarding the relationship between environmental conservation and food innovation. More work is needed in this area to discover the reasons behind the skepticism and to further assess the impact of food technology and innovation on environmental protection and preservation in the context of organic foods. 

## Figures and Tables

**Table 1:** Respondents’ demographic information.

Demographic Factor	Options	Malaysia		Hungary	
		Freq.	Percentage (%)	Freq.	Percentage (%)
Gender	Male	123 (16.820 *)	41.0 (51.45)	224 (4.680 *)	60.2 (47.91)
	Female	177 (15.880 *)	59.0 (48.55)	148 (5.088 *)	39.8 (52.09)
Age	Below 20	65	21.7	4	1.1
	21–40	214	71.3	137	36.8
	41–50	13	4.3	123	33.1
	51–60	4	1.3	87	23.4
	Above 60	4	1.3	21	5.6
Marital Status	Single	249	83	108	29
	Married with children	46	15.3	198	53.2
	Married without children	3	1	28	7.5
	Single with children	2	0.7	20	5.4

Note: * Numbers in bracket represent national populations in millions. The Hungarian population statistics are obtained from [74]. The Malaysian population statistics are obtained from [75].

**Table 2:** Research variables and indicators with mean and standard deviation.

Research Variables	Indicators	Malaysia		Hungary	
		Mean	SD	Mean	SD
Health Consciousness (HC) Yang et al. [76]; Shaharudin et al. [52]	HC1—Healthy diet is an important factor when choosing what I eat	4.230	0.775	3.867	0.786
	HC2—I give a lot of attention to my health	3.826	0.837	4.11	0.745
	HC3—A healthy body is important to me	4.421	0.626	3.045	0.993
	HC4—Health concern is the reason for consuming organic food	3.919	0.874	3.140	1.00
	HC5—Proper nutrition is a key factor for purchasing organic food	3.909	0.848	4.196	0.909
Environmental Concern (EC) Yang et al. [76]	EC1—I am concerned about the state of our environment	3.993	0.798	3.457	1.065
	EC2—Environmental concerns affects my food choice	3.692	0.926	3.370	1.165
	EC3—Organic food is environmentally friendly	3.916	0.876	3.869	1.103
	EC4—Chemical fertilizers are harmful for the environment	4.143	0.910	4.382	0.8663
	EC5—Everyone should be concerned for our environment	4.568	0.707	3.471	1.067
Perceived Quality (PQ) Aulia et al. [58]	PQ1—Organic food is a healthier food option	4.220	0.788	3.353	1.078
	PQ2—Organic food has great nutritional benefits	4.153	0.880	3.251	1.059
	PQ3—Organic food has better quality due to its advanced cultivation methods	4.016	0.849	3.252	1.104
	PQ4—Though I may have to pay more, I get better quality organic food	3.879	0.926	3.225	0.9942
	PQ5—I am satisfied with organic food quality	3.923	0.939	3.733	0.9866
Social Lifestyle (SL) Basha et al. [57]; Falguera et al. [65]	SL1—Organic food is a trend in society	3.493	1.01	2.175	1.104
	SL2—My family influence me to consume organic food	3.177	1.14	2.046	1.036
	SL3—My peers influence me to consume organic food	2.959	1.04	2.754	1.22
	SL4—Celebrities often promote organic food consumption	3.214	1.09	3.807	0.935
	SL5—The lifestyle of consuming organic food is healthy	3.953	0.861	3.549	0.9742
Food Innovation Adoption (FIA) Self-Developed for this Study	FIA1—The way organic food is grown and processed influence me to consume organic food	3.721	0.908	2.843	1.132
	FIA2—The advantages of GM (genetically modified) foods outweighs potential disadvantages	3.476	0.931	3.495	1.036
	FIA3—Advances in food technologies have produced better quality food for the world	3.845	0.825	3.769	0.8930
	FIA4—Technologically superior organic food production improves food yields	3.815	0.860	3.939	0.9062
	FIA5—Innovation in food production is to be welcomed by all	3.922	0.796	3.877	0.9858
	FIA6—I support technology and innovation in food production	4.000	0.794	2.695	1.155
Consumer Purchase Intention Towards Organic Food (CP) Shaharudin et al. [52]	CP1—I purchase organic food frequently	2.966	1.12	2.587	1.143
	CP2—I will continue to purchase organic food	3.391	0.985	2.791	1.165
	CP3—I am willing to pay more for organic food than conventional food in the store	3.351	1.03	2.887	1.186
	CP4—I will recommend organic food to family and friends	3.738	0.918	2.195	1.194
	CP5—I consider myself a loyal organic food consumer	3.023	1.24	3.34	1.011

**Table 3:** Internal consistency, composite reliability and convergent validity.

Variable	Indicator	Factor Loadings		Cronbach’s Alpha		Composite Reliability		AVE	
		MD	HD	MD	HD	MD	HD	MD	HD
Health Consciousness (HC)	HC1	0.754	0.627	0.786	0.725	0.860	0.821	0.607	0.537
	HC2	0.768	0.655						
	HC3	0.650	0.809						
	HC4	0.801	0.820						
	HC5	0.720	-						
Environmental Concern (EC)	EC1	0.707	0.684	0.749	0.729	0.841	0.825	0.570	0.546
	EC2	0.720	0.815						
	EC3	0.766	0.607						
	EC4	0.635	-						
	EC5	0.767	0.826						
Perceived Quality (PQ)	PQ1	0.778	0.863	0.828	0.888	0.885	0.922	0.660	0.748
	PQ2	0.780	0.873						
	PQ3	0.817	0.890						
	PQ4	0.797	0.832						
	PQ5	0.843	-						
Social Lifestyle (SL)	SL1	-	0.673	0.673	0.671	0.787	0.752	0.515	0.513
	SL2	0.799	0.652						
	SL3	0.712	0.773						
	SL4	0.553	0.665						
Food Innovation Adoption (FIA)	FIA1	0.696	-	0.813	0.724	0.876	0.753	0.640	0.524
	FIA2	0.725	0.554						
	FIA3	0.739	-						
	FIA4	0.817	0.600						
	FIA5	0.798	0.603						
	FIA6	0.750	0.845						
Consumer Purchase Intention Towards Organic Food (CP)	CP1	0.847	0.919	0.896	0.914	0.923	0.919	0.706	0.796
	CP2	0.841	0.883						
	CP3	0.830	0.904						
	CP4	0.805	0.861						
	CP5	0.878	-						

Note: MD stands for ‘Malaysian Data’ (*n* = 300); HD stands for ‘Hungarian Data’ (*n* = 372).

**Table 4:** The Heterotrait-Monotrait ratio of correlations (HTMT).

Malaysian Data						Hungarian Data					
	HC	EC	PQ	SL	FIA		HC	EC	PQ	SL	FIA
HC						HC					
EC	0.7260					EC	0.6272				
PQ	0.7212	0.7012				PQ	0.5968	0.8143			
SL	0.5757	0.3903	0.7851			SL	0.5162	0.7971	0.7720		
FIA	0.6335	0.6632	0.6631	0.6067		FIA	0.4229	0.5024	0.5512	0.5965	
CP	0.5099	0.3945	0.6405	0.6544	0.5104	CP	0.6738	0.7666	0.6357	0.8427	0.5773

**Table 5:** Results of hypotheses testing for Malaysia and Hungary.

Hypothesis	Relationship	Malaysian Data		Hungarian Data	
		Path Coef.	*p*-Value	Path Coef.	*p*-Value
H1	HC → CP	0.600	0.107	0.185	<0.001 ***
H2	EC → CP	−0.011	0.860	0.117	0.014 **
H3	PQ → CP	0.271	<0.001 ***	0.187	0.002 ***
H4	SL → CP	0.306	<0.001 ***	0.113	<0.013 **
H5	FIA → CP	0.109	0.035 **	0.414	<0.001 ***
H6	HC → FIA	0.110	0.0402 **	0.188	<0.001 ***
H7	EC → FIA	0.341	<0.001 ***	0.014	0.852
H8	PQ → FIA	0.134	0.036 **	0.313	0.031 **
H9	SL → FIA	0.187	<0.001 ***	0.329	<0.001 ***

*** Significant at 1%; ** Significant at 5%

**Table 6:** Indirect effects of factors towards CP through FIA.

Hypothesis	Relationship	MD		HD	
		Path Coef.	*p*-Value	Path Coef.	*p*-Value
H10	HC → FIA → CP	0.012	0.253	0.078	0.003 **
H11	EC → FIA → CP	0.037	0.082 *	−0.004	0.890
H12	PQ → FIA → CP	0.014	0.212	0.129	<0.001 **
H13	SL → FIA → CP	0.020	0.057 *	0.136	<0.001 **

** Significant at 1%; * Significant at 10%

**Table 7:** Visual representation of Path Modelling Results showing the relative importance of the constructs.

Constructs	Malaysian Consumers		Hungarian Consumers	
	Food Innovation Adoption	Organic Food Purchase	Food Innovation Adoption	Organic Food Purchase
Health Consciousness	Important (4)		Important (3)	Important (3)
Environmental Concern	Important (1)			Important (4)
Perceived Quality	Important (3)	Important (2)	Important (2)	Important (2)
Social Lifestyle	Important (2)	Important (1)	Important (1)	Important (5)
Food Innovation Adoption		Important (3)		Important (1)

Note: Numbers in bracket show the ranking and relative importance of factors within the column.
